# Net benefit of smaller human populations to environmental integrity and individual health and wellbeing

**DOI:** 10.3389/fpubh.2024.1339933

**Published:** 2024-03-05

**Authors:** Chitra Maharani Saraswati, Melinda A. Judge, Lewis J. Z. Weeda, Quique Bassat, Ndola Prata, Peter N. Le Souëf, Corey J. A. Bradshaw

**Affiliations:** ^1^Telethon Kids Institute, Perth, WA, Australia; ^2^School of Mathematics and Statistics, University of Western Australia, Nedlands, WA, Australia; ^3^School of Medicine, University of Western Australia, Nedlands, WA, Australia; ^4^ISGlobal, Hospital Clínic - Universitat de Barcelona, Barcelona, Spain; ^5^Centro de Investigação em Saúde de Manhiça (CISM), Maputo, Mozambique; ^6^Catalan Institution for Research and Advanced Studies (ICREA), Barcelona, Spain; ^7^Paediatrics Department, Hospital Sant Joan de Déu, Universitat de Barcelona, Esplugues, Barcelona, Spain; ^8^Centro de Investigación Biomédica en Red (CIBER) de Epidemiología y Salud Pública, Instituto de Salud Carlos III, Madrid, Spain; ^9^Bixby Center for Population Health and Sustainability, School of Public Health, University of California, Berkeley, Berkeley, CA, United States; ^10^Global Ecology | Partuyarta Ngadluku Wardli Kuu, College of Science and Engineering, Flinders University, Adelaide, SA, Australia; ^11^Australian Research Council Centre of Excellence for Australian Biodiversity and Heritage, Wollongong, NSW, Australia

**Keywords:** air pollution, child health, climate change, consumption, environment, overshoot, pediatrics, sustainability

## Abstract

**Introduction:**

The global human population is still growing such that our collective enterprise is driving environmental catastrophe. Despite a decline in average population growth rate, we are still experiencing the highest annual increase of global human population size in the history of our species—averaging an additional 84 million people per year since 1990. No review to date has accumulated the available evidence describing the associations between increasing population and environmental decline, nor solutions for mitigating the problems arising.

**Methods:**

We summarize the available evidence of the relationships between human population size and growth and environmental integrity, human prosperity and wellbeing, and climate change. We used *PubMed*, *Google Scholar*, and *Web of Science* to identify all relevant peer-reviewed and gray-literature sources examining the consequences of human population size and growth on the biosphere. We reviewed papers describing and quantifying the risks associated with population growth, especially relating to climate change.

**Results:**

These risks are global in scale, such as greenhouse-gas emissions, climate disruption, pollution, loss of biodiversity, and spread of disease—all potentially catastrophic for human standards of living, health, and general wellbeing. The trends increasing the risks of global population growth are country development, demographics, maternal education, access to family planning, and child and maternal health.

**Conclusion:**

Support for nations still going through a demographic transition is required to ensure progress occurs within planetary boundaries and promotes equity and human rights. Ensuring the wellbeing for all under this aim itself will lower population growth and further promote environmental sustainability.

## 1 Introduction

Growth of the global human population is one important dimension of the rising severity of climate change, but is often not overtly discussed as a driver. For example, the Sixth Assessment Report by the Intergovernmental Panel on Climate Change (IPCC) did not mention population in its widely disseminated *Summary for Policymakers* (1), although population was discussed in the full report (2). Neither was population mentioned in either the *Paris COP 21 Agreement* (3) or the *Glasgow COP 26 Climate Pact* (4). The reason for this lack of emphasis on the contribution of population growth to environmental decline, including climate change, is unclear, but it possibly stems from sensitivities regarding unclear messaging (5), inequalities between high- and low-income nations, and concerns about challenging the established paradigm that economic growth is necessary for development (6). If the issue of the human contribution to environmental integrity and future wellbeing are to be given proper consideration and discussed rationally, it is essential that the population morass be included in any debates.

The effects of climate change on human health have been the focus of extensive research, but the contribution of population growth to these effects have been largely overlooked. This oversight threatens to diminish recent improvements in global health. Although the global fertility rate is slowly declining, the annual rate of population increase relative to planetary boundaries has not changed in 30 years, with the annual increment exceeding 80 million (7). The contribution of population increase to environmental integrity and resilience remains one of the greatest gaps in understanding. Finding acceptable and ethical solutions to the quandary of population in terms of maintaining resources and human health and wellbeing is therefore urgent. Emphasizing that women and men globally have access to free, non-coercive, culturally and socially acceptable, and high-quality family-planning services is an important component of these solutions in the long term. Indeed, this is one of the United Nations Sustainable Development Goals, but is still a long way from being met (8).

In this review, we summarize the available evidence of the relationships between human population size and growth and environmental integrity, human prosperity and wellbeing, and of course, climate change. After revealing the available evidence, we suggest approaches to mitigate negative repercussions. We review the broad range of ways in which a high and increasing population contributes to increasing consumption, rising emissions, and continuing environmental damage. Given that climate change is the greatest threat to future human health and persistence (2), including the potential to interact with other socio-economic drivers exacerbating conflict (9), we examine the contribution of an increasing population to this threat, including the potential of overshooting current population projections. We examine the evidence for the impact of environmental change, including climate change, on human health, with an emphasis on child health given that 88% of the climate-change health burden is borne by children (10, 11). Finally, we examine the arguments for and against policies to stimulate or reduce population growth globally, nationally, and locally.

We have organized our review into the following sections: (*i*) introduction (*ii*) basics of population projections, how these measures are created, and potential limitations to be considered from existing global population projections, (*iii*) risks from increasing global population size, where we consider the implications of the highest population growth projections (“worst case” scenario), (*iv*) drivers of increasing risk of population overshoot, (*v*) countering arguments against a safe and sustainable global population, specifically addressing the unfounded fears associated with population decline and aging populations, and (*vi*) discussing potential policy pathways to achieve safe and sustainable population sizes globally.

## 2 Materials and methods

We employed a search strategy in *Pubmed*, *Google Scholar*, and *Web of Science* to identify all relevant peer-reviewed and gray-literature sources examining the consequences of human population size and growth on the biosphere. Our main search terms included: “population,” “demography,” “fertility,” “overpopulation,” “population size,” “family planning,” “projection,” and these expanded rapidly to incorporate elements associated with “climate change,” “greenhouse-gas emissions,” “consumption,” “ecological footprint,” “biocapacity,” “pollution,” “biodiversity,” “disease,” “contraception,” “child health,” “maternal education,” and “population decline.” We also followed many additional pathways identified via these search strings to online reports and databases to complete the coverage of available literature. To determine the trends in peer-reviewed publications addressing the joint topics of population and human fertility, we employed the search string “population + fertility” in *PubMed* from 01.01.1970 to 31.12.2022.

## 3 Results

### 3.1 Current population projections and risk of overshoot

#### 3.1.1 Human population size and projected trends

**Figure 1:**
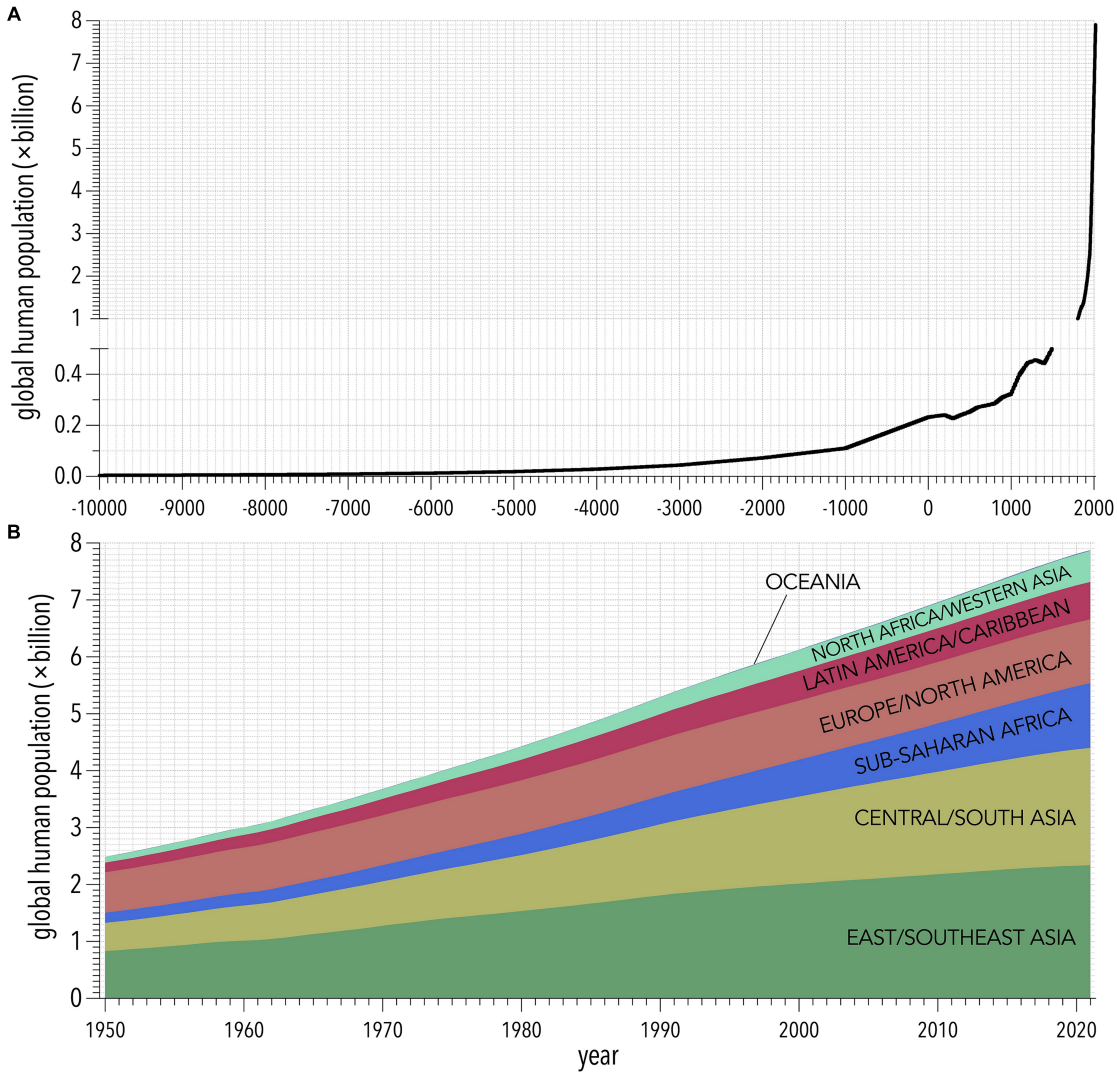
**(A)** Population growth since 10,000 BCE according to the History Database of the Global Environment and the United Nations (12). **(B)** Regional population trajectories from 1950 to 2021 from the United Nations Population Division (7).

There are now over eight billion people living on Earth (12). The world population has increased at an unprecedented rate since the 1700s (Figure 1), and is projected to increase to an average of 10.4 billion people in 2100—a 10-fold increase over 250 years (7).

Several different models project the population trajectory to 2100. The most widely cited are from the World Population Prospects produced by the Population Division of the Department of Economic and Social Affairs of the United Nations Secretariat (7). The United Nations Population Division projections have been updated regularly since 1951, and therefore have broad appeal. Alternatively, forecasts from the International Institute for Applied Systems Analysis-Wittgenstein Centre (IIASA) (13), available since the 1990s, are the most widely used for modeling the contribution of future emissions in climate-change projections (2). Another more recent forecast (from 2020) is by the International Health Metrics and Evaluation (IHME) (14), but with criticism of its methods and results (15, 16).

All population projections have the same starting point: estimates of the number of people alive today tend to be consistent among models, with birth and mortality rates derived from censuses, demographic surveys, or official registers. The differences in projected population size derive from different modeling choices and assumptions when applying estimates of fertility, mortality, and international migration parameters. Some of the main differences in projected outcomes depend on the expectation of how fertility, mortality, and migration will change with anticipated economic development, as well as how quickly each country might progress through the demographic transition—the theory (17) proposing how nations move from high fertility prior to a decline, followed by a rapid decline in fertility, to plateau eventually at a low fertility.

United Nations Population Division projections use the cohort-component method (18), where existing population dynamics are constructed for each country and projected to 2100. Future survival probabilities, future number of births, and future net migration are projected in five-year intervals using nine projection variants, with five of these variants differing in fertility assumptions (low, medium, high, constant fertility, or instant-replacement fertility), but assuming constant mortality and net migration. The other four variants assume medium fertility but vary mortality and net migration. The 2022 report projects the global population to peak at 10.4 billion people in the 2080s and to remain there until 2100 under its medium variant, and assumes that total fertility rates will continue to decline (7). The lowest-rate variant projects the global population to decline to 8.9 billion by 2100, and the highest-rate variant projects it to rise to 12.4 billion, with this variability arising from an uncertain projection of fertility rates (7). The increasing frequency of pandemics (19) might add uncertainty to forecasts of fertility rate due to the accompanying pattern of a steep initial decline in fertility, followed by gradual increases and a baby boom (20).

The IIASA forecasts take educational attainment into account, in addition to the conventional age and sex structures (13, 21), to project populations in three scenarios based on shared socio-economic pathways derived from both expert opinions and statistical modeling. The *Medium* scenario forecasts a medium pathway for fertility and mortality rates, generally viewed as the most likely from today’s perspective. The *Rapid Social Development* scenario assumes rapid increases in life expectancy, a faster decline of fertility rates in currently high-fertility countries, and a fulfillment of the education goals in the United Nations’ Sustainability Development Goals. The *Stalled Social Development* scenario assumes a stall in education attainment within developing countries, and continued high fertility and mortality. The 2018 *Medium* projection predicts a global population of 9.8 billion achieved between 2070 and 2080 before slowly declining to 9.5 billion people by 2100. In the *Rapid Social Development* scenario, a peak population of 8.9 billion is projected for 2055–2060 before declining to 7.8 billion by 2100. Assuming the *Stalled Social Development* conditions, the world population is forecasted to be 10 billion people in 2045, with a continued growth to 13.4 billion by 2100.

The main difference between the IHME projections and those from the United Nations Population Division and IIASA is the quantification of fertility. Instead of the conventional total fertility rate, defined by the World Health Organization as the “… total number of children that a hypothetical cohort of women would have at the end of their reproductive period if they were subject during their whole lives to the fertility rates of a given period and if they were not subject to mortality” (22), the IHME instead applies the completed cohort fertility at age 50 (CCF50), defined as the “… average number of children born to an individual female from an observed birth cohort if she lived to the end of her reproductive lifespan” (14). The CCF50 has been proposed as a more stable forecasting method because it corrects for changes in total fertility rate over time rather than assuming the raw values that fluctuate considerably over time, due to lags in the influence of changing age structure, educational attainment, and meeting contraceptive needs. While using CCF50 might improve the stability of projections, larger influences on variation among models are the specific assumptions regarding the trajectories of future fertility, education, age structure, and other development indicators. The IMHE projections consider four alternative scenarios with differences in education and family-planning policies. De-prioritizing education and family planning through policy changes increased projected population sizes. The lowest-rate forecast assumed increased female empowerment through better education attainment and increased access to contraception (14), resulting in a lower growth rate than the *Medium* variant of the United Nations Population Division projections—this projects the global population to peak at 9.73 billion just after mid-century and then a decline to 8.79 billion people by 2100 (14).

Accurate population projections are an important tool in shaping the future of human societies primarily through their effects on national policies—for example, planning for health care, housing, childcare, and schools, or anticipating economic development (15, 16, 23). We discuss the interaction between projections of population size and these policy dimensions below. Of the three projections highlighted, the IHME’s has been most criticized for proposing less-realistic and least-verifiable assumptions (24). The corollary is that unsubstantiated lower projections to 2100 could potentially mislead governments to implement coercive policies such as restricting access to contraception to increase fertility to a replacement rate. The argument for this is usually driven by a misplaced fear of stagnation in the country’s economy, the arguments against which we discuss in subsequent sections.

There is a lack of rigorous evaluation of existing population projections in terms of relative assumptions and realism of proposed scenarios. A necessary analysis of existing projections under various scenarios would clarify the relative likelihood of different population trajectories over the course of the coming century. While the three institutions responsible for the described projections include working groups of experts, an external and independent evaluation would guide future improvements and provide more realism. Regardless, the most likely outcomes based on mid-range assumptions and scenarios indicate that a global population between 9 and > 10 billion by the end of this century is the most parsimonious.

### 3.2 Risks from increasing population sizes

#### 3.2.1 Consequences of a growing human population

Large human populations pose a risk of global catastrophe due to their influence on increasing environmental risks such as changes in atmosphere and climate, land degradation, and threats to biodiversity. Fundamentally, continued population growth leads to an increase in human economic activity, which puts pressure on the planet’s ability to renew resources (25). Population growth increases pressure and competition for finite resources such as food, water, and land; to compensate, production must rise, resulting in even greater environmental damage (26–28). With fewer people in the past, whenever environmental damage occurred, groups of people usually colonized other places or otherwise survived at lower densities (25). With today’s already large global population, the option to colonize new regions is unfeasible and instead drives additional environmental damage (26, 28). The causes for these global environmental risks are not necessarily clear, so we consider the following main environmental problems arising from population growth: (*i*) greenhouse-gas emissions and temperature increases, (*ii*) pollution, (*iii*) loss of biodiversity, and (*iv*) spread of infectious diseases and general worsening health outcomes.

There is a strong theoretical basis for expecting a positive relationship between human population size and the risk of environmental erosion that are encapsulated by several mathematical concepts. Existing models built to quantify environmental impact arising from population pressures, such as the IPAT (**I**mpact [emissions] = **P**opulation × **A**ffluence × **T**echnology) (29), ImPACT (**I**mpact = **P**opulation × **A**ffluence × **C**onsumption [intensity of use] × **E**fficiency [emissions per unit energy used]) (30), and STIRPAT (a stochastic variant of IPAT) (31), were developed mainly to determine the role of factors such as population growth and technological change in affecting environmental degradation. These equations and their variants can ideally predict the environmental outcomes of particular policy adjustments (32, 33). For example, the ImPACT equation was constructed to assess total emissions as a function of population size, *per capita* gross domestic product, energy consumption per unit gross domestic product, and CO_2_ emissions per unit energy consumption (30). The following subsections reveal the extent to which the population component of impact equations cannot be neglected.

#### 3.2.2 Greenhouse-gas emissions and climate disruption

It is axiomatic that an increasing population produces more emissions, but this simple relationship belies a complex interplay between consumption rate itself, and the total number of consumers. We explore this concept contextually before considering the consumption and production pathways resulting in different emissions profiles.

**Figure 2:**
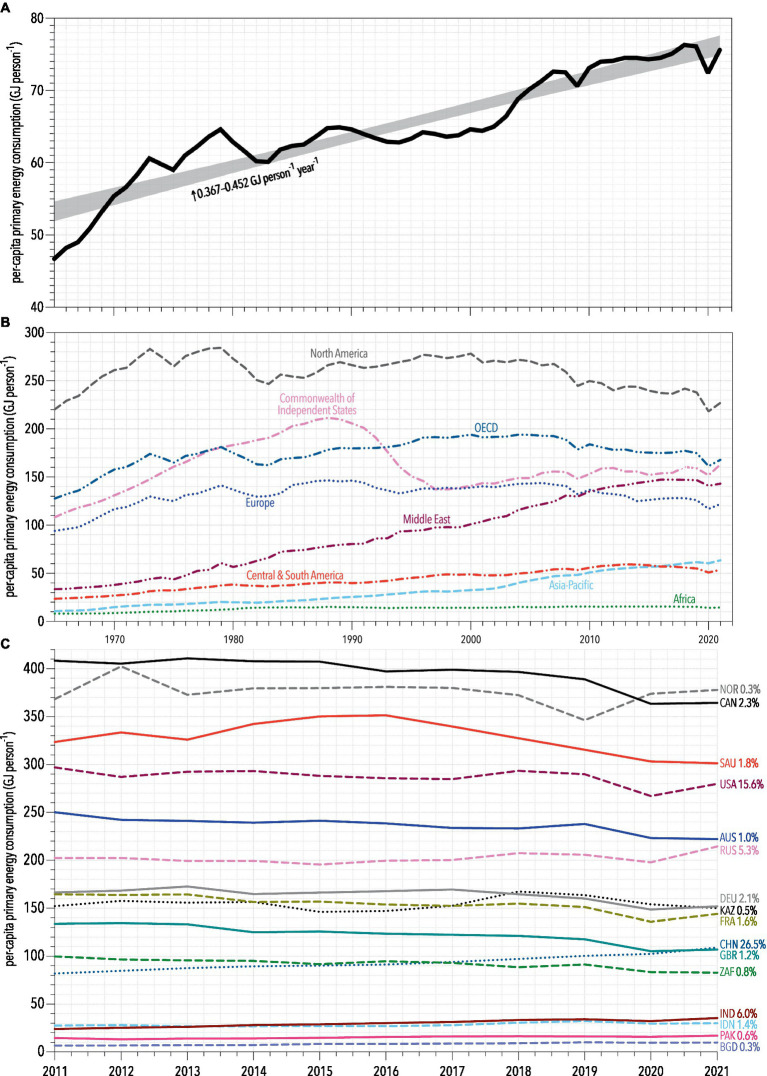
**(A)** Per-capita annual primary energy consumption from 1965 to 2022 (36), equating to an average increase of 0.41 GJ person^−1^ year^−1^ (95% confidence interval: 0.367–0.452 GJ person^−1^ year^−1^). **(B)** Regional per-capita annual primary energy consumption over the same interval. **(C)** Trajectories of per-capita primary energy consumption from example countries covering the broad range. Percentages next to the country abbreviations indicate the share of total global consumption in 2021 (36). Countries shown: NOR, Norway; CAN, Canada; SAU, Saudi Arabia; USA, United States; AUS, Australia; RUS, Russia; DEU, Germany; KAZ, Kazakhstan; FRA, France; CHN, China; GBR, United Kingdom; ZAF, South Africa; IND, India; IDN, Indonesia; PAK, Pakistan; BGD, Bangladesh.

Increased concentrations of greenhouse gases generated from energy consumption, predominantly from burning fossil fuels, have contributed greatly to global environmental degradation. The Intergovernmental Panel on Climate Change (IPCC) Assessment Reports show that increasing emissions translate to additional global warming, reduced air quality, changes in the global water cycle, and increased prevalence of extreme climate events such as high rainfall and flooding, fires, droughts, and cyclones (34), with all the associated secondary adverse health impacts. Population growth has resulted in more human enterprise, and therefore, more intensive energy consumption (35). As new technology is developed, the energy consumption *per capita* also grows (27, 28). Indeed, the annual per-capita rate of global primary energy consumption increased 1.62 times since 1965 to today (46.7–75.6 GJ person^−1^), or an average increase of 0.41 GJ person^−1^ year^−1^ (Figure 2) (36). During that same interval, the global human population increased from 3.3 to 8.0 billion (Figure 1) and total annual greenhouse-gas emissions increased from 11.2 Gt to 33.0 Gt CO_2_-e (36).

A combination of more consumers and higher consumption rates drive the growth in greenhouse-gas emissions, rather than population growth alone (37). Nations with low per-capita emissions tend to have the highest population growth rates (38), meaning that if they follow the development trajectories of high-income nations today, emissions will also continue to grow. The latest IPCC report predicts a 1.7°C increase in global temperatures relative to pre-industrial temperatures (i.e., average of the 51-year period from 1850 to 1900) by 2060 under a scenario of low population growth versus 2.8°C warming with a medium-high scenario of population growth (34, 39).

**Figure 3:**
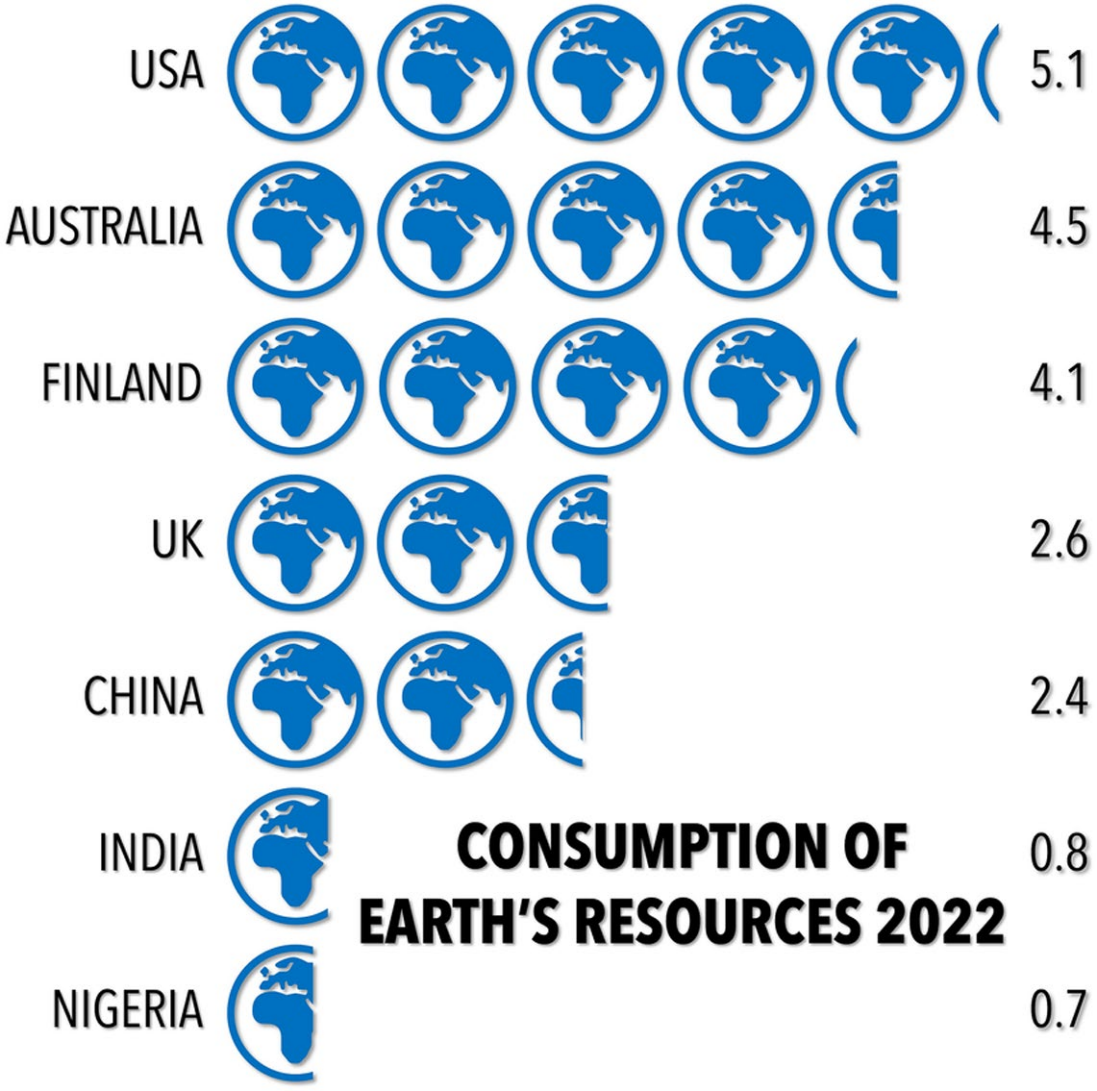
Our global ecological footprint using seven example nations: USA, Australia, Finland, United Kingdom, China, India, and Nigeria (41, 42). The numbers on the right indicate how many Earths would be required if everyone on Earth consumed renewable resources at the same rate as the nation indicated.

Consistently throughout history, there is a concomitant rise in per-capita energy consumption with population growth (35). As people gradually deplete resources in their environment, innovators find new ways to extract energy from previously unused resources or import resources from less-depleted locations. Increased energy use from these new resources facilitates improvements in diet and living standards, which stimulates even more population growth (26, 35, 40). This phenomenon is summarized succinctly by the concept of the *ecological footprint*, which describes how much biologically productive area is required to provide for all the competing demands of the people it services, such as space for agricultural and fiber production, timber, sequestration of carbon dioxide emitted from burning fossil fuels, and built infrastructure (41) (Figure 3). This area can be calculated for the entire globe, or individual nations, leading to the estimate of *biocapacity*, which is the amount of biologically productive land and sea available to provide the resources a particular population consumes and to absorb its wastes (given current technology and management). Globally, we are operating on a biocapacity deficit that is consuming Earth’s ecosystems 1.7 times more rapidly than they can be renewed.

Greater consumption of manufactured goods increases waste and subsequently leads to increased consumption of resources to manage that waste, creating a self-perpetuating, vicious cycle. Food-waste management alone accounts for 8–10% of global human-produced greenhouse-gas emissions year^−1^ (approximately half of that emitted by the entire global food system) (43, 44). Solid waste management adds another 5% (45). Carbon-removal technologies such as carbon capture, carbon sequestering, and other proposed “net zero” solutions will not themselves counter increasing consumption. While appealing in principle, these technologies are logistically difficult to implement at scale such that net emissions decline. Relying on such technologies is dangerous because it diminishes the sense of urgency to reduce consumption and emissions now (46).

Population growth is the main driver of water scarcity because larger populations have higher water demand (47, 48) in the absence of major technological and policy shifts to disrupt the dependency of demand and supply (49, 50). Freshwater resources are finite, but the demand for water in food production continues to grow. Irrigated agriculture, rain-fed agriculture, and livestock production on pastureland all require freshwater. Subsequently, population growth exacerbates environmental risks by requiring greater food production (40, 51).

The agriculture sector contributes over a quarter of the world’s greenhouse-gas emissions, from agriculture, forestry, and land-use change (52–55). Agriculture accounts for approximately 20% of global, human-produced greenhouse-gas emissions annually (55), while livestock contributes 14.5% (56). Food production emits 45% of total methane (CH_4_) globally, where 80% is from livestock production, and rice production is the next-largest emitter. Agriculture is also responsible for 80% of nitrous oxide (N_2_O) emissions globally, mainly from fertilizer application (57). Methane and nitrous oxide are more powerful than carbon dioxide (CO_2_) in forcing temperature increases over a span of 20 years by a multiple of 84 times and 264 times (55), respectively. Together, CO_2_, CH_4_ and N_2_O concentrations have increased over the industrial era from human activity, resulting in unequivocal warming of the global climate system (34).

Reducing emissions in agriculture is challenging because the sector (*i*) is slower to change than other major industries, (*ii*) is fragmented, and (*iii*) has a complicated set of objectives. Unlike the electricity sector, where it is possible to displace coal and gas with low-emissions technologies (58, 59), these options are not available for agriculture. Another contributing deterrent of rapid, broad-scale change is the large proportion of small stakeholders. Most farmers (2 billion globally) are employed on small farms (smallholders) in developing countries (60), meaning 65% of working adults in low-income countries make a living through agriculture. The risk of failure or lower yields in the short term is therefore untenable despite potential long-term gains for reducing emissions at the farm scale (60). Most emissions-reduction measures, such as more sustainable farming practices, would either reduce costs or be cost-neutral; however, they are not implemented due to capital constraints, limited access to technology, and adherence to local traditional practices, all exacerbated by the scale of smallholdings (53). Additionally, agriculture impacts biodiversity preservation, nutritional needs, food security, and livelihoods. Forests in developing countries are, on average, cleared twice as fast as they can grow back (61), leading to the concern that soil erosion and desertification from deforestation combined with intensive agriculture threatens up to a third of the Earth’s total land surface. Financial support and capacity building for smallholders are essential to bring the agricultural sector to a more sustainable path and to fulfill its goals of reducing emissions.

As the human population continues to increase, awareness of what we eat and how much food we waste (consumption-side management) is essential. Managing food waste is the most impactful because approximately one-third of all food produced is never consumed (62). Wastage occurs across the supply chain during production, transportation, and storage due to lack of access to technology and cold-storage infrastructure. It also occurs at the retail and consumption phases, especially in higher-income regions due to aesthetic preferences, purchasing more food than needed, and poor portion control. If food waste were to fall to <30% by 2030 and < 20% by 2050, there would be an overall reduction in greenhouse-gas emissions from food waste by about 40% globally (62).

Although a much smaller effect, consumption should be considered when reducing the sector’s emissions. As people become wealthier, demand for meat consumption tends to grow (63). High-income countries consume between 60 and 91 g day^−1^ of meat, while countries in Asia and Africa consume only 4–7 and 7–34 g day^−1^, respectively (64). Meat consumption and production are environmentally costly; food systems are responsible for 21–37% of global greenhouse gas emissions, and of that, 52% is caused by cattle products alone (65).

The common denominator for all these issues is population growth. Despite implementing the solutions proposed above specific to each outlined problem, if the global population keeps growing beyond a safe space for the planet, we will still experience disastrous consequences. Indeed, the most effective individual action in addressing the emission and consumption issue is to have one fewer child (66).

#### 3.2.3 Pollution

Despite ongoing efforts by United Nations agencies, committed groups and individuals, and some national governments (mostly in high-income countries), little real progress against both air and water pollution is being made overall, particularly in the low-income and middle-income countries where pollution impacts are most severe (67). Deaths from ambient air pollution have increased over the last two decades, accounting for 2.9 million premature deaths in 2000, increasing to 4.2 million in 2015, and 4.5 million in 2019 (67). Premature deaths due to all forms of pollution have remained unchanged at 9 million from 2015 to 2019 (67). *The Lancet Commission on Pollution and Health* cited pollution as “… the world’s largest environmental risk factor for disease and premature death” (67).

There is also a growing concern for water quality—population pressure, unsustainable consumption, and unsustainable production stress can degrade freshwater resources (47). In the past, smaller settlements relied on the self-cleansing and dilution potential of rivers when disposing effluent. These natural functions reach their limits with greater population density and increased industrial production, calling for increased regulation of effluent disposal (47).

More waste resulting from an increase in consumption of manufactured goods also increases pollution, with low- and middle-income countries disproportionately burdened by environmental destruction through pollution due to inadequate infrastructure for waste management—up to 93% of all waste in low-income countries is dumped without further processing (45). This poor management of waste through open dumping or uncontrolled burning pollutes soil, water (68), and the air, subsequently reducing crop growth (69), increasing water scarcity, and damaging human health (45).

#### 3.2.4 Loss of biodiversity

Resource extraction beyond the regenerative potential of Earth is responsible for biodiversity loss, but work remains to identify the relative impact of different mechanisms, and temporal and spatial scales, of the degradation ensuing. The challenge is teasing apart the effects of consumption *per se*, and overall population size—as in all forms of anthropogenic damage to the biosphere, the product of consumption and number of consumers is the combined driver of biodiversity loss. Currently, an annihilation of Earth’s biodiversity is underway because of human endeavor, such that we are now firmly within the sixth mass extinction event (70, 71).

An increase in human population size is generally correlated with worse outcomes for biodiversity health in protected areas (72). Human population density and growth rates are disproportionately higher in Biodiversity Hotspots (areas with exceptionally high species endemism and concomitant threats from human agency) (73, 74), which subsequently leads to higher deforestation rates and species loss (75). Historically, an increase in human population size is associated with greater threats to biodiversity (76, 77), and strongly associated with an increased number of threatened species (78–80). Factors contributing to species threat include habitat destruction and degradation (71), direct exploitation such as hunting (81), invasive species (82–84), pollution (85, 86), diseases (87, 88), climate change (89, 90), and the synergies emerging from these different drivers (91).

Another component of biodiversity loss is land-use conversion for human activities such as agriculture, mining, logging, establishing transport networks, and urbanization. Eight times as much temperate grassland is converted for human purposes than is protected (92). Agriculture is the largest driver of biodiversity loss worldwide (52, 93), with a third of the world’s land surface already converted for agriculture (94, 95), and over half of the world’s wetlands drained and repurposed for agriculture in the last century (96, 97). The global livestock sector is rapidly growing and intensifying, with livestock usually displacing local fauna (56, 98). The expansion of plantations and pastoralism since the 1980s has resulted in tropical deforestation (99, 100). Large herbivore and carnivore species on land also have declining populations due to agriculture (101–103); for large carnivores such as lions, most are pre-emptively hunted because they are threats to humans and livestock (104, 105). In the ocean, examples of destruction of the environment include overfishing (106), trawlers destroying ocean habitats (107–109), and the extinction of large fish species (110). The indirect driver of all this destruction is population growth as we ramp up agricultural production to keep pace; the World Resources Institute has estimated that we need to close a 56% food gap between calories produced (as of 2010) and those needed in 2050 if the global population was to rise to 10 billion people (111).

#### 3.2.5 Spread of disease

Increasing human population means more people living in urban areas (from 43% in 1990 to 54% in 2015) (112). If population growth continues at a similar rate, around 68% of all people will reside in urban communities by 2050, with most urbanization occurring in African countries (112). Rapid urbanization underlies an increasing prevalence of non-communicable diseases in low- and middle-income countries, which account for 85% of premature deaths (between the ages of 30 and 69 years) from noncommunicable diseases worldwide (113). The most prevalent of these include cardiovascular disease, cancers, chronic respiratory disease, and diabetes, all of which have common behavioral risk factors such as poor diet, limited exercise, smoking, and drinking alcohol (114, 115). Additionally, people with a non-communicable disease are at increased risk of some infectious diseases such as tuberculosis or COVID-19, or experience worse health outcomes, such as antiretroviral therapy-treated people living with HIV infection (114). This burden of disease is a threat to economic development by affecting the productivity of working-age people (114).

Urbanization affects the young through a reduction in fertility rates and reduced risk of child undernutrition (115, 116), but an increased risk of becoming overweight (115). Modeled outcomes from 73 countries showed that while children living in urban slums have better health outcomes than rural children, they are not as good as children living in better-off urban environments (116). Urban children, both poor and rich, have reduced mortality and stunting compared to rural children, but increased recent episodes of illness (116).

The associations between urbanization and infectious disease are many and complicated, leading to either increased or reduced risk depending on context (112, 115). Rapid urbanization is strongly linked to informal settlements (slums) that lack basic infrastructure (water and sewerage access) and are overcrowded (higher population density), which can heighten the spread of infectious pathogens (112). Yet, urban communities can provide more accessible health-care facilities and socio-economic opportunities that can lead to improved health outcomes (115). Urban risk factors encompass two main groups: (*i*) geographic, including population density, the built environment, municipal services, and the natural environment; and (*ii*) behavioral, including hygiene and sanitation, education and employment, sexual behaviors, and socioeconomic conditions (112).

With rising urbanization comes increased population density and higher prevalence or transmission of infectious diseases including tuberculosis (117, 118), yellow fever (119), Ebola (120), and HIV (121, 122), particularly in slums. Even within households, risk increases directly with household size (112). Human-to-human disease transmission increases largely due to close contact, while high population density can expose more people to vectors of disease. For example, despite highly effective mosquito-control programs in the high-income nation of Singapore, human population growth and rapid urbanization have enabled far fewer mosquitos to infect the overcrowded population and increase the prevalence of dengue fever (123). Furthermore, as global warming extends the length of the transmission season of mosquito-borne diseases, urban communities will be disproportionately burdened (124). Climate change poses a novel situation in which vector-borne diseases are able to be introduced to and survive in immunologically naïve populations (125). This is reflected in dengue becoming the most rapidly spreading mosquito-borne disease worldwide, with a 30-fold increase over that last 50 years as climates on the fringes of tropical and subtropical regions change to facilitate its spread (126). Within Australia, this is mirrored in migration and increasing burden of Murray Valley viral encephalitis and Ross River virus (127).

The built environment has differing impacts on the risk of infectious disease in humans. Irregularly or sparsely built-up urban areas have higher malaria risk, while densely built areas closer to the city center have reduced malaria risk (128). However, the magnitude of this disparity depends on localized environmental conditions such as proximity to dense vegetation, bodies of water (hydrographic network (128), artificial lakes and dams), or swampy areas, which all increase malaria risk (129). The risk of dengue fever is magnified in urban slums through inadequate drinking water, rubbish collection, and drainage of surface water, leading to increased mosquito breeding and consequent pathogen transmission (130). Conversely, improved health-care access in urban areas compared to rural can improve health outcomes. Malaria is an example of an infectious disease whose expansion has been aided by climate change, global temperature rise in particular. A 1°C increase in mean and minimum temperatures in Nepal led to a 27% incidence increase of malaria countrywide and 25% increase in geographical regions impacted by the disease (131). Likewise, warming is pushing upwards the maximum elevation of malaria in the highlands of countries like Ethiopia and Colombia (132).

Multiple aspects of the quality of house-building have been implicated in the prevalence of infectious disease, with irregularly or poorly built homes associated with increases in respiratory diseases, malaria, and helminth infections (112). Furthermore, rapid urbanization often goes hand in hand with a lack of municipal services such as hygiene (rubbish collection and waste management/disposal), sanitation, and healthcare services, which greatly increase the risk of some infectious diseases. A lack of household latrine and drainage systems has been associated with increased incidence of cholera (133, 134), bacterial and protozoal enteric infections (135), and diarrhea in children (136).

Higher population densities in urban areas can directly increase the risk of poor health outcomes. Increasing urbanization across Africa in particular has been associated with more deaths from air pollution as countries develop economically with increasing industrialization (67). While factors of the natural environment such as wetness and temperature can increase the risk of infectious diseases in urban and rural areas alike, population density in urban areas can increase transmission (112, 133, 137).

Zoonotic transmission of diseases accounts for an estimated 61% of human pathogens and 75% of pathogens that are deemed emerging (138). The occurrence of zoonotic transmission in regions experiencing rapid urbanization is becoming commonplace with expanding consequences. The severe acute respiratory syndrome (SARS) outbreak in 2003 (139), H1N1 influenza pandemic of 2009, Middle Eastern respiratory syndrome (MERS) outbreak in 2012, and of course, the COVID-19 pandemic, are well-documented diseases that have all been facilitated by rapid urbanization (140). Migration and travel both between rural and urban areas, and more globally, can swiftly disseminate disease. Proximity of an urban community to some animal populations, exacerbated by deforestation, forest degradation, and biodiversity loss more generally (141), can have profound impacts on the epidemiology of infectious diseases. Waste accumulation from human habitation encourages rodents and stray animals, plus water storage can enable mosquito proliferation, both of which can increase the chance of spreading a zoonotic disease in urban areas (142). Emergence or re-emergence of infectious diseases through increased interaction at the wildlife-livestock-human interface, at areas of steep transition between ecosystems (known as “ecotones”), can increase the likelihood of disease transmission between species. This is confounded by climate change and biodiversity collapse, both associated with large population sizes, which increase the risk of pathogen exchange at the human-animal interface (125). Evidence of disease emergence at ecotones has been documented for yellow fever, Nipah virus encephalitis, influenza, rabies, hantavirus pulmonary syndrome, Lyme disease, cholera, *Escherichia coli* infection, and African trypanosomiasis (143). Land-use change, particularly deforestation, has increased proximity of humans with wildlife directly, or through livestock that interact with wildlife (142). Livestock production that overuses and misuses antibiotics can increase antibiotic-resistant bacterial strains, which are transferable to humans through direct contact with infected animals, consumption of contaminated food, or via the environment (water, soil, air) contaminated by animal waste (144).

**Figure 4:**
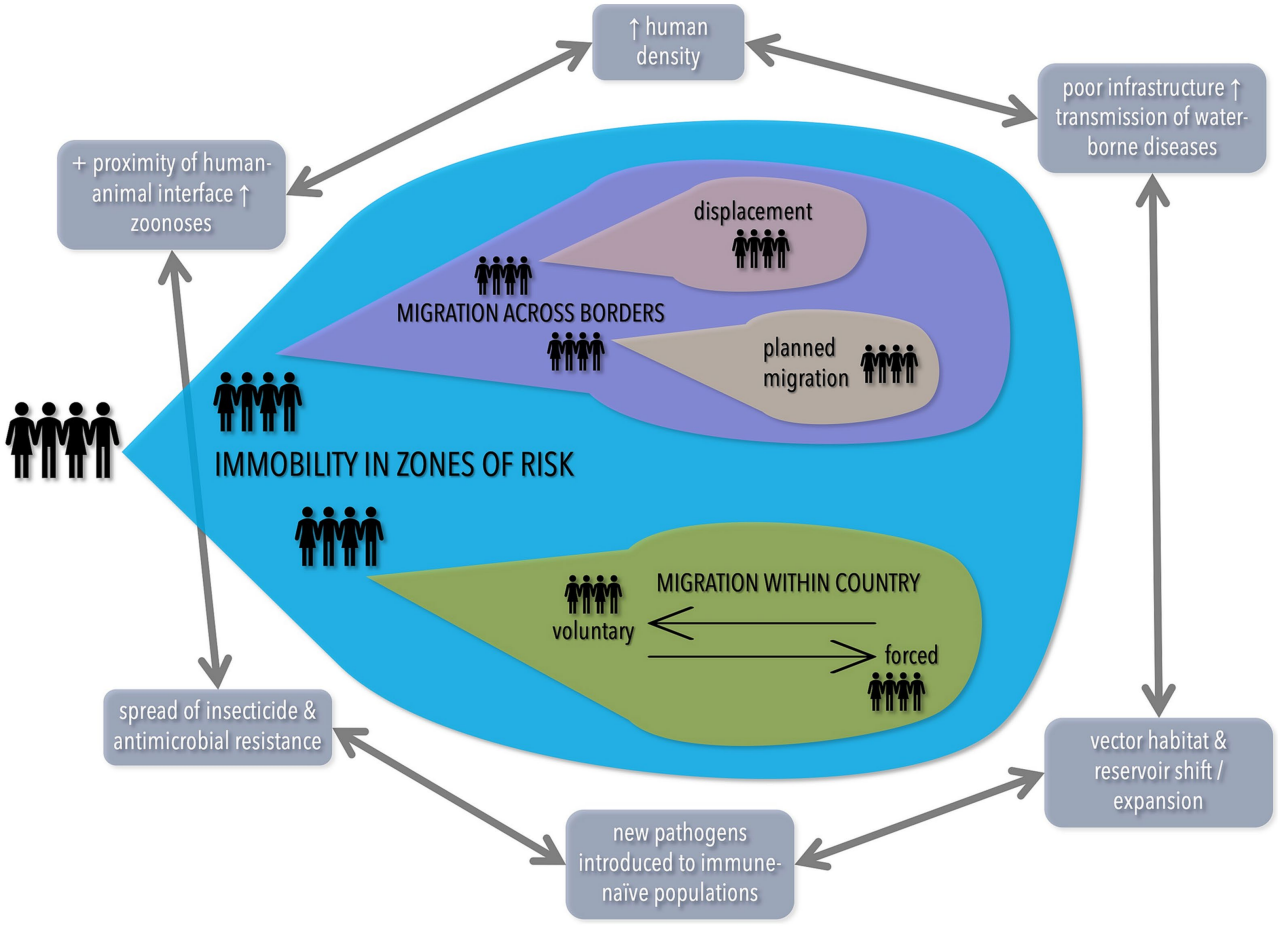
Population density, climate change, and poor infrastructure/planning all interact to lead to increased risk of disease. Adapted with permission from Wiley (Ref. 125), © 2021 Paediatrics and Child Health Division (The Royal Australasian College of Physicians), https://doi.org/10.1111/jpc.15681.

Rapid urbanization often occurs without adequate planning, leading to more violence, conflict, and crime. This burden disproportionately affects women, migrants, and refugees, with impacts on security, livelihoods, health and access to services (145) (Figure 4).

Increasing prevalence of urbanization can be expected to continue and can be beneficial to delivery of health services if accompanied by informed planning and policy. The faster urbanization occurs (with a rapidly increasing world population), the less planning will happen (slums emerge much faster than planned urban developments, especially in sub-Saharan Africa) and therefore, more of the negative effects of urbanization are likely. This is complicated by climate change that increases the prevalence of many infectious and non-infectious diseases (125). In short, the 2021 Australian State of the Environment report stated it best: “Environmental degradation is now considered a threat to humanity, which could bring about societal collapses” (146).

### 3.3 Risk of overshoot

**Figure 5:**
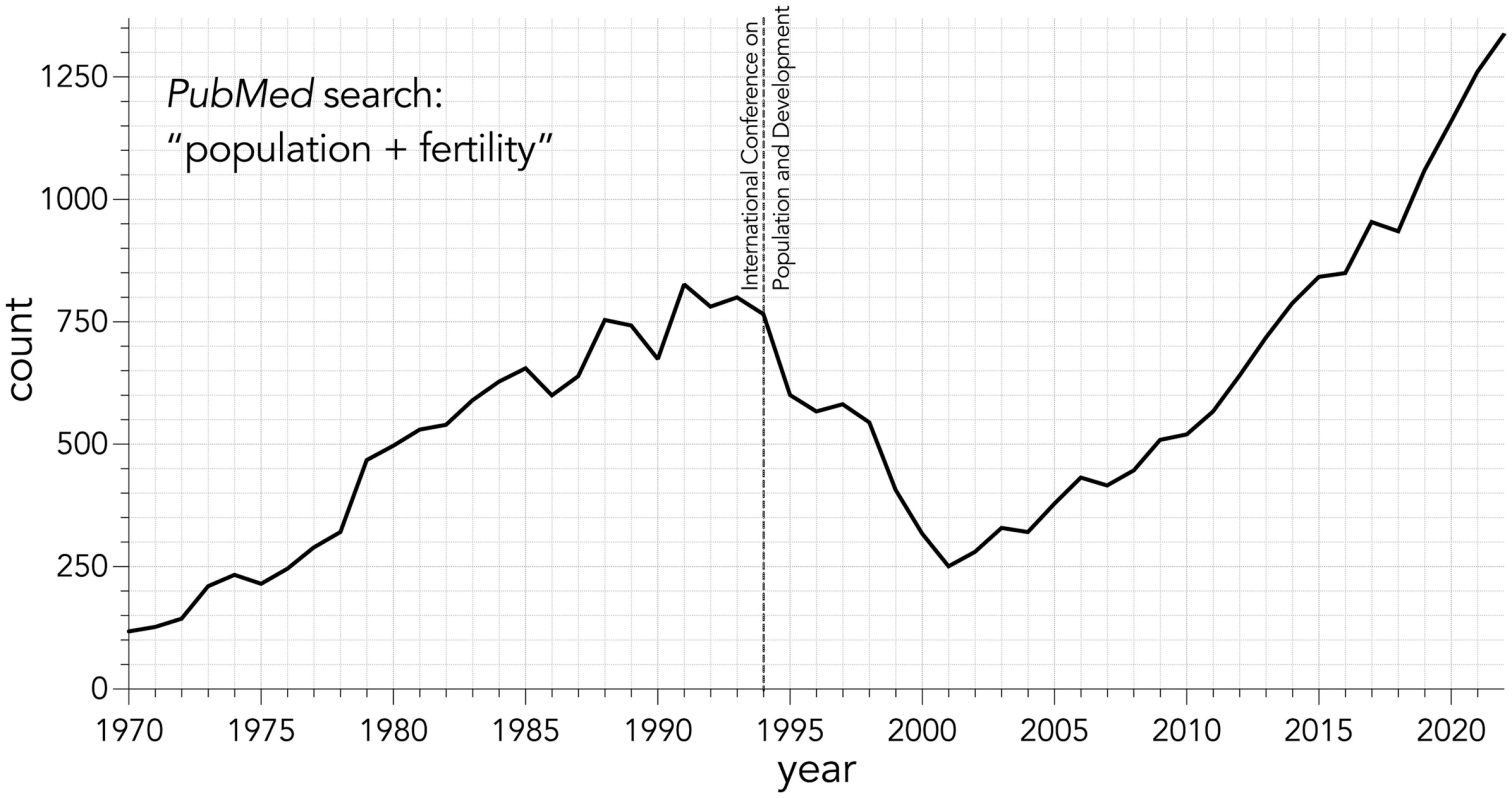
Annual total number of articles identified from *PubMed* using the search string “population + fertility” from 1970 to 2022.

The steady rise of publications investigating human fertility and population that we identified using a search of the online engine *PubMed* (date range 01.01.1970 to 31.12.2022) dwindled sharply following the landmark 1994 International Conference on Population and Development held in Cairo (32) (Figure 5). That conference sparked a pivotal change in international discussions regarding population from the starting perspective of global population “control” through increased access and quality of family planning, to an individual-based model focused on improving the rights of women and girls through access to education and reproductive health services (33, 147). However, the meeting was dominated by voices from the Vatican and their views around contraception and abortion, which denuded discussion of topics such as the environmental impacts of population growth. This remains an ongoing issue because international policy discussions today still stifle the conversation on population. The United Nations Sustainable Development Goals do not mention slowing population growth, with only one Goal (3.7 *Good Health and Wellbeing*) mentioning universal access to sexual and reproductive health-care services (148). This lack of prioritization is further demonstrated because the role of population in international policies today is analyzed by a subsidiary group of the United Nations (United Nations Population Fund), who are supported only through voluntary contributions from governments and not through a regular budget (149).

Projections of population increase are inherently unreliable because the ultimate expression of future population depends heavily on even small changes in total fertility rate per country (see section 3.1.1). Intelligent discussion on overpopulation has also been inhibited due to concerns of “population control” related to past abuses arising from autocratic measures to limit fertility (150). Providing women and men the opportunity to determine the number and spacing of their children, free of coercion is not, by definition, “population control,” and was internationally recognized as a basic human right in 1968 (151). In particular, empowering women, especially disadvantaged women, to be able to make decisions about when and how many children they intend to have, improves their own lives as well as those of their children, and is a proven path to successful development (152). In 2006, only around half of the world’s population lived in countries with fertility rates at or below replacement, notably only in high-income countries (153).

Below we discuss some of the factors that lead to women choosing to have more children, thereby increasing fertility rates. There are many influences on a woman’s total fertility rate; we distill these into the following main categories: (*i*) demographic drivers, (*ii*) lack of access to contraceptives, (*iii*) child and maternal health, (*iv*) maternal education, and (*v*) social and cultural factors. We also discuss the principle that determining the optimal, case-by-case family size should not be left to women alone; men should also be provided with the education and free access to effective male contraception, allowing them to contribute actively in family-planning decisions.

#### 3.3.1 Demography

The age at which women first give birth has a large impact on fertility rates, with an average younger age at first birth reducing the intergenerational gap, and increasing fertility rates over time (153). For example, if all women in a society started having children at the age of 20 years as opposed to 25, the population would be at least 20% larger in 100 years (assuming other factors remain unchanged) (154, 155). However, exceptions to this pattern have been observed within East Africa and Afghanistan, where subnationally, the highest teenage fertility rates do not always correspond with the highest fertility rates (152). This indicates the influence of additional behaviors and social norms, bearing in mind that East Africa and Afghanistan have some of the highest total fertility rates globally (152). Conversely, older average maternal age at first birth reduces reproductive lifespan, producing a lower average number of total children and lowering fertility rates overall (153, 156). Population (or “demographic”) *momentum*, a natural consequence of the demographic transition (154), where high fertility rates of the previous generation increase population growth in the current generation even when fertility rates are declining (157), is another contributor to higher population growth rates (158).

#### 3.3.2 Lack of access to contraceptives and unintended pregnancies

An estimated 270 million women had an unmet need for family planning in 2019 (159), 85 million of which used traditional rather than modern methods of contraception (160). This number rose from 232 million in 1990 and is expected to rise to 272 million by 2030, mainly due to family-planning services in developing countries not keeping pace with the rapid population increase (159). Globally, approximately half of all pregnancies in 2015–2019 were unintended, which equates to 121 million unintended pregnancies annually (161). There is a strong inverse relationship between unintended pregnancy and World Bank income group, with sub-Saharan Africa experiencing the highest rate of unintended pregnancy, and Europe and North America, the lowest (161). Not every unintended pregnancy is unwanted; however, an estimated 61% of unintended pregnancies end in abortion, totaling 73.3 million abortions annually (161). In countries where abortion is restricted, the proportion of unintended pregnancies that end in abortion has increased since the early 1990s, and their rates of unintended pregnancy were higher than in countries where abortion was legal (161).

The post-Cairo framing on women’s rights primarily had an unintentional negative impact of taking the focus off access to family planning, and thus led to some governments deprioritizing family planning. This has occurred recently, with the UK government cutting 85% of its annual funding to the United Nations Population Fund (162). Domestic politics can also play a large role on global family planning services; for example, the major global funder, the United States Agency for International Development (USAID) has precluded the provision of abortion since 1984 under the “global gag rule” by anyone receiving those funds (163), depending on the sitting US president.

Access to contraceptives and non-coercive, quality family-planning services are mechanisms to help populations from attaining a size that generally reduces the standard of living, health, and wellbeing. Indeed, in sub-Saharan Africa where large families are common, the availability and quality of family-planning services had the largest effect on fertility of any explanatory variable (0.83 fewer births per woman) (164). However, subsequent research demonstrates that most variation in fertility among low- and middle-income nations can be explained by variation in child mortality, followed by household size (a proxy for population density), and then access to any form of contraception (165). That family-planning services educate parents about the benefits of investing in fewer children has been observed previously (164).

In addition to lowering fertility, family planning also improves the health of mothers and children. Contraceptive use, by reducing the number of births, therefore reduces the number of times a woman is exposed to birth-related mortality risks, and also reduces the incidence of problems arising from high-risk, high-parity births (166). Maternal mortality remains the leading cause of death and disability in reproductive-age women in low- and middle-income countries (167), with one study estimating over 1 million maternal deaths were averted between 1990 and 2005 in low- and middle-income countries through access to contraception (166). In 2008, an estimated 342,203 women worldwide died of maternal causes, with contraceptive use averting 272,040 deaths (preventing 44% of probable mortality), and if the unmet need for contraception was satisfied, another 104,000 maternal deaths could have been avoided (29%) (168). In Indonesia, contraceptive use averted an estimated 523,885–663,146 maternal deaths between 1970 and 2017 (169).

Contraceptives and other family-planning services allow women to modify the risks that come with pregnancies that are “… too early, too late, too many, or too frequent” (170). Shorter birth intervals were associated with higher infant and child mortality in a large longitudinal study in Bangladesh (171), thereby supporting the maternal depletion hypothesis where high fertility does not allow a woman to recuperate sufficiently from the nutrient/energy depletion caused by the first pregnancy or breastfeeding event to support a subsequent pregnancy (171). Longer birth intervals can increase the probability of nutrition repletion, which can positively affect fetal growth and newborn survival, although the results are equivocal among studies (172). Birth spacing of >24 months reduced the probability of child stunting in Indigenous communities of India, with increased access to family planning suggested as a major intervention to enable improved child health (173). Other mechanisms that might influence mortality risk of a short birth interval include sibling competition for parental time and resources, maternal wellbeing, and increased risk of disease transmission among similarly aged siblings (171). Furthermore, a short birth interval reduces infant survival (174, 175), thereby simultaneously increasing the woman’s probability of having another child, and reducing the time to the next birth (consistent with “replacement” behavior (176), whereby infant death truncates breastfeeding and reduces protection against fertility) (177).

When a young mother dies, there are cascading effects beyond the motherless infant. In 1990, 585,000 women died from pregnancy-related causes, leaving behind at least 1 million motherless children (170) who have twice the risk of dying compared to children whose father had died only, and daughters almost twice as likely to die compared to sons (170). Similarly, an Ethiopian study concluded that a maternal death imposed an increased chance of the infant dying 46 times higher than if the mother had survived (167). The HIV/AIDS epidemic has resulted in approximately 17 million children who lost one or both parents, with 90% of those children living in sub-Saharan Africa (178), and devastating consequences for individuals and communities (179). Given the dire consequences, it is surprising that access to safe, effective, affordable, and acceptable family-planning services has not improved since the 1994 Cairo meeting. In response, the 2012 London Summit on Family Planning developed goals for improved access, which have not yet been met (180).

Despite the overall stalling of family planning globally, there are successful examples in low- and middle-income nations due to political will and government leadership. Between 2005 and 2015, the Rwandan Government expanded and promoted family planning, increasing the use of contraceptives from 17 to 53% (181). Similarly, the Ugandan Government also recognized the immediate need for access to family planning and has pledged to increase funding (182), given that rapid population growth coupled with a high young-age dependency ratio (more young people than working-age people) is economically unsustainable and will prevent Uganda attaining middle-income status (182). Policy implemented over 5 years has already provided 1.5 million women with family-planning services and averted 8,000 maternal and 100,000 child deaths, and saved over US$300 million in pregnancy-related health-care costs.

#### 3.3.3 Child health

Infant and child mortality has declined rapidly, with global infant mortality moving from 98.5 deaths/1000 live births in 1970 to 27.9 deaths/1000 live births in 2021 (i.e., a 3.5-fold reduction) (7). However, this impressive reduction belies high regional variability, with low- and middle-income countries disproportionately concentrating up to 99% of the world’s child mortality (sub-Saharan Africa 49 deaths/1000 live births, and South Asia 30 deaths/1000 live births) as of 2021 (7). Additionally, both climate change and the continuing rapid increase in population are expected to limit the rate of future mortality reduction. A complex and multifaceted relationship exists between population pressures, climate change, and child health. Because these aspects interlink in unique and often poorly understood ways, the exploration of this topic can easily become misdirected and overwhelming. Relationships are also confounded by social, economic, and geographical contexts, exemplified by considering two children from vastly different socio-economic-geographical backgrounds who both face the implications of overpopulation. A child who lives in an environment with poor access to healthcare, limited economic opportunities, and a governmental/political system limiting her ability to live healthily will be at a much greater risk of overpopulation-associated issues, such as malnutrition and decreased food security. Conversely, a child living in a country with greater socio-economic-health opportunities will be better equipped to deal with the pressures of a high-population and climate-disrupted future.

**Figure 6:**
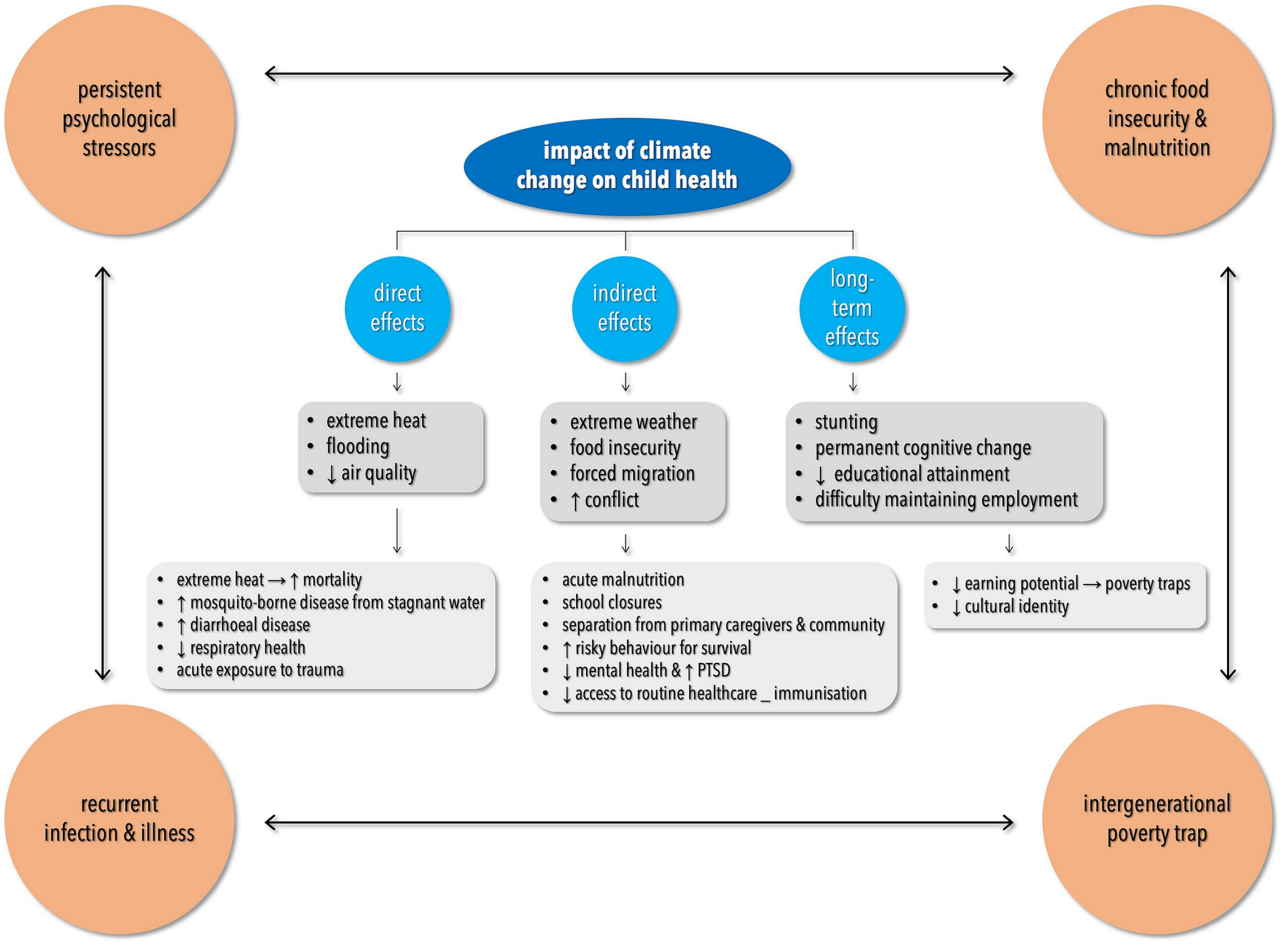
Climate change has direct, indirect, and long-term impacts on child health that are influenced by socio-economic and geographical conditions. Adapted with permission from Wiley (Ref. 183), © 2021 Paediatrics and Child Health Division (The Royal Australasian College of Physicians), https://doi.org/10.1111/jpc.15704.

The literature on overpopulation and child health can be broadly categorized into (*i*) direct impacts, (*ii*) indirect impacts, and (*iii*) examination of physical and behavioral changes resulting from child-health status (Figure 6). Direct impacts of overpopulation on child health include the ways in which overpopulation exacerbates food insecurity, malnutrition, and therefore, poor health outcomes. The indirect impacts include more varied mechanisms often concerned with how overpopulation drives climate change that impedes child health. The ecological footprint concept (41) demonstrates that the combination of population and consumption outstrip the planet’s ability to sustain our collective behavior. Based on United Nations data from 2005 to 2007, approximately 800 million people globally are undernourished, and food requirements will need to increase by 40% by 2030 and 70% by 2050 to maintain this proportion of malnourishment (184). But food security is threatened by an increasing population straining vulnerable food-supply systems and by a changing climate damaging and limiting food production itself (185, 186). The geographical distribution of the Earth’s undernourished population is mainly in Asia (381 million undernourished) and Africa (250 million undernourished) (8) where overpopulation exacerbates the problem (187) and is centered on large nuclear families. Having limited economic resources in large families reduces nutrition and healthcare in children (187); therefore, overpopulation threatens child health by placing strain on economic resources.

Poor food security that begets malnutrition is not only underscored by limited economic capacity, but also by threats to the food-supply network, with climate change being one of the greatest threats operating mainly via increasing drought (186). Data based on the approximately 20% of malnourished children aged <5 years old in northern Kenya demonstrate that drought raises this percentage incrementally (188). In Ethiopia, a 1°C rise in average prenatal temperature was associated with a 28% increase in the odds of severe stunting in early life (189). Temperature increases can reduce crop yields, thereby restricting nutrition including for many pregnant women, resulting in lower birth-weight offspring and an increased prevalence of stunting and child mortality (190). In the Ethiopian context, an exception to this phenomenon exists in the cooler highlands, where a temperature increase often decreases frost damage to crops and consequently increases food security (189). Climate change alters other aspects of weather systems beyond drought. In Indonesia, a 40-day delay in the monsoon can reduce rice yield by 6.5–11% (191), while a 44-day delay led to a 6.3% decline in mean height-for-age in children (192). Such accumulative, short-term disruptions translate to losses in long-term nutritional status, regardless of the local region’s ability to recover from such an event.

Climate change affects almost every aspect of our environment, and so also affects human physiology and health, with children being one of the most vulnerable age groups (193). In fact, children will bear 88% of all health adverse consequences related to climate change given their unique physiological and behavioral characteristics, in addition to accumulated exposure (194). In New York, increased heat variability from climate change increased the prevalence of pediatric presentation to hospital (195). In the Northern Territory of Australia, temperature extremes lead to increased pneumonia presentations to hospital, especially for children (196). In both California (197) and Europe (198), heat is a risk factor for respiratory admissions, yet the causal mechanism is not well-understood. Extreme temperatures have also been associated with other undesirable health outcomes in children, namely low birth weight (199), stunting (189), low Apgar scores (200), and increased risk of stillbirth (201). Increasing humidity in wet seasons promotes transmission of respiratory infectious disease in both Brazil (202) and Indonesia (203).

Climate change-exacerbated air pollution threatens child health. Childhood exposure to oxidants (O_3_ and NO_2_) are associated with increased incidence of asthma and eczema (204), and early exposure to increased pollutant concentrations trigger atopic dermatitis in children (205). In Italy, a 10-grain m^−3^ rise in total aeroallergen concentration increased the risk of asthma presentation to hospital not only on the day of the event, but also 2 days afterwards (206). Bushfires are becoming more frequent due to climate change (186), producing air pollutants with detrimental health impacts. For example, a 1 μg m^−3^ increase in the concentration of fire-related PM_2.5_ is associated with a 2.17-g reduction in birth weight (207), and increases in fire-sourced air pollutants have been linked with increased risk of pregnancy loss (208).

Of the known effects of climate change on child health, preterm birth (209) is the best-described. Regions at the highest risk of preterm birth due to extreme heat are those with colder and drier climates (210). In Minnesota, USA, pregnant women exposed to a 7-day heatwave of >37°C faced greater risk of preterm birth (211). In China, pregnant women are at greatest risk of preterm delivery when exposed to extreme heat during the third trimester (212), and in Spain preterm birth risk increased up to 20% when maximum temperature exceeded the 90th percentile over the 2 days prior to delivery (213). Similar results abound in many other regions of the world—e.g., Belgium (214), Australia (215), and Israel (209). Thus, while overpopulation threatens child health directly, it also drives anthropogenic climate change that, in itself, degrades child health (216).

#### 3.3.4 Maternal education

The effect of maternal education on human fertility is complicated and equivocal depending on which aspects of “education” are measured, and the scale of investigation. Within nations, there is evidence that higher female education lowers fertility; for example, in Nigeria each additional year of education reduced fertility by 0.26 births/woman on average, as well as increased the age at primiparity (217, 218). Likewise, data from Ethiopia, Kenya, Tanzania, and Zimbabwe revealed that fertility fell most, and birthing interval increased most, among women with secondary education from the 1970s to the 2000s (219).

At broader spatial scales (among-country), the influence of maternal education on fertility is more ambiguous. Based on Demographic and Health Survey data from 43 countries, increasing educational attainment correlated with lower fertilities (220). The most-accepted paradigm—based on ample time-series data from single countries; e.g., Brazil (221), Kenya (222), Bangladesh (223), India (224)—is that child mortality declines as a mother’s years of education increases, thereby de-incentivizing families to have more children. However, a more recent study examining data for 64 low- and middle-income nations revealed that while child mortality was the strongest predictor of variation in fertility, female education attainment (years of education completed) did not provide any additional explanatory power (165). However, it remains unclear whether education, while providing increased autonomy, is most responsible for the reduction in fertility *per se* (222), instead of the ability to seek medical interventions, the socioeconomic impact of higher-income employment, or a high-income earning husband. While strongly correlated, the link might not be causal, with maternal education acting more as a proxy for socio-economic status and geographic area of residence (225).

#### 3.3.5 Social and cultural influences

Fertility is sequential, time-limited, and non-reversible, with fertility rate varying as a function of *tempo* components (i.e., age at primiparity, birth intervals) and *quantum* (e.g., whether parents can afford a large family, name continuation, potential contribution to household economies) (226). Thus, several other dimensions dictate fertility trends beyond education, infant mortality, and access to family planning. For example, a study of 70 low- and middle-income countries with high-fertility clusters determined that while low female secondary education attainment, low contraceptive use, and high unmet need for family planning were partially responsible (152), it also identified high-fertility clusters in areas crossing country borders, suggesting an influence of local cultural values rather than country-specific family-planning policies (152). Urbanization itself has been linked to lower fertility, but the quality of modernization arising from urbanization (e.g., economic opportunities) is an important element modifying the expected relationship (227). Some cultures also emphasize sons over daughters. This can manifest as shorter birth intervals when the previous child was a daughter (228), or the higher likelihood of opting for a “replacement” birth following the death of a son compared to a daughter (229).

Governments also introduce policies that affect population growth. In 1978 in China, statisticians and economists determined that population growth had to be reduced to reach the aim of quadrupling the per-capita national income, thereby laying the foundation for the one-child policy (230). Despite reaching the goal of reduced population growth, the violation of human rights was abhorrent. Similar results might have been achieved through the provision of quality, non-coercive, culturally appropriate family-planning services. Conversely, other parts of the world are currently experiencing cultural and religious barriers undermining women’s hard-won rights to exercise choice. In 2020, the USA reduced funding and access to family planning services (231, 232), and Russia has recently prioritized “population growth” as a top health priority (233). Hungary and Poland, two countries with a history of restricting women’s rights under conservative governments (234, 235), have near-total bans on abortions, reducing access in recent years (236–238).

### 3.4 Concerns about aging and declining populations

#### 3.4.1 Population decline

Concerns (239–242) about population decline are rooted primarily in fears of an associated economic decline, with a potential reduction in gross domestic product a commonly used argument. Here, a population decline is assumed to lower the number of working adults, subsequently lowering productivity, and thus lowering national gross domestic product. The arguments against the validity of brute measures of market activity as reasonable indices of national wealth notwithstanding (243), a decline in gross domestic product is proposed to reduce innovation and lead to economic and fiscal challenges; indeed, traditional economic thought sees population growth as a major source of economic growth (241, 244). Lower growth of gross domestic production might also be driven by a reduction in domestic consumption as older people are thought to purchase fewer consumer durables than younger people. Another identified concern ensuing from low economic growth is a shift in geopolitical power as currently, higher gross domestic product is associated with higher geopolitical power. Other potential issues of a declining population include complexities in fiscal policies such as national health insurance and social security (245). Fears surround a contracting working population being burdened by an expanding aging population (246).

These concerns ignore existing evidence regarding the many economic and wellbeing advantages of smaller populations. First, the fear of population decline ignores that none of the existing population projections (see section 3.1.1) predict a decline in the global population (7, 13, 14). The global population is still growing (Figure 1), so the possibility of a “population collapse” over the coming century is nil. Second, stated concerns inherently assume a reduction in gross domestic product is a negative outcome, yet economic models show this indicator does not necessarily measure wellbeing, either for individuals or the planet (247). Continued growth in gross domestic product is an unconstrained, capitalist, pro-growth view that is not sustainable. Neither do lower fertility rates themselves imply lower economic growth. In fact, reduced fertility can increase capital per worker and per-capita consumption provided by human capital investments (248, 249). Lower fertility rates are also proposed to increase income *per capita* and lower carbon emissions through changes in total population size, age structure, and economic output (250). Assumed negative impacts also make unsupported assumptions about the continuation of past productivity trends, which are themselves mitigated by developments in technology. It is therefore difficult to quantify the potential effect of technology on future economic growth, because technology can also buffer change via low-cost labor supply (251).

More importantly, lower populations provide environmental advantages. Indeed, the available evidence shows that only 25% of the increase in greenhouse-gas emissions globally is attributable to per-capita increases in consumption, whereas 75% is due to population growth (252, 253). However, the IPCC Climate Change Synthesis Report Summary for Policymakers (1) does not mention population growth as a major diver of climate change. A decrease in population growth would reduce global emissions provided that consumption decreases at a comparable rate in the short term, but should promote large emission reductions in the long term. If the unmet need for family planning was filled, global emissions could be reduced by an estimated 0.7–1.25 Gt of carbon year^−1^, or approximately 8–15% of the reduction in emissions needed to avoid warming of >2°C by 2050 (254–256). Based on projections from the United Nations 2004 World Population Prospects (7.4, 8.9, and 10.6 billion by 2050 for the low, medium, and high scenarios, and 5.5, 9.1, and 14.0 billion by 2100, respectively) (257), the low-growth path would reduce emissions by 1.4 Gt year^−1^ by 2050 (−15%) and 5.1 Gt year^−1^ by 2100 (−40%) compared to the medium path (258). In contrast, the high-growth path would increase global emissions by 1.7 Gt year^−1^ by 2050 (+17%) and 7.3 Gt year^−1^ by 2100 (+60%) compared to the medium path (258). While many assumptions underlie these estimates (e.g., economic growth trends, technological shifts, energy transitions, population structure, urbanization), they do not take resource constraints or environmental degradation limiting population growth into account (257). For example, urbanization alone is expected to increase projected emissions by >25%, especially in the case of developing regions (259). However, urban living is more energy efficient than rural living after controlling for income, which can cause a net decrease in emissions (259). Additionally, rapid urbanization can hasten the transition to cleaner fuels (260, 261).

Alternatively, population growth is potentially disadvantageous to a country’s economy if it cannot keep up with the rising number of people to employ youth productively (262). For example, Angola’s population growth rate of 3% year^−1^ since 1970 increased the population of 6 million to 33 million today—one of the world’s highest rates of annual population growth (263, 264). Accompanying this growth is its poverty rate that increased by 15% between 2008 and 2018 (265). Angola’s youth today suffer from poor living standards that its government and economy are unable to alleviate (266).

Even those economists purporting “profound social and economic implications” (246) state that the transition to older societies in a few countries (e.g., Japan) is “manageable” via structural reforms, technological advances, and debt stabilization. No financial “crisis” is on the horizon, but there will be a requirement to adjust existing fiscal policies, including to health systems and retirement funding (245). Furthermore, such adjustments are entirely realistic in the low-corruption, high rule-of-law countries where aging populations are of concern.

In conclusion, a downward trajectory of fertility and population growth rates is in our collective interest. There is no evidence that a lower population growth rate is necessarily detrimental to an economy.

#### 3.4.2 Aging populations

A rise in the global aging population is another argument raised to promote population growth. The concerns mirror those stated for population declines: labor shortages, increased government expenditure in health care and pension funds, and reduced consumption—all culminating in economic decline (239–242). Labor shortages are feared to drive up prices and lower living standards. The proportion of persons aged ≥65 years globally is indeed projected to rise from 10% in 2022 to 16% in 2050 (7). This changing age structure will create some demographic challenges, as existing economic and fiscal policies will need to be restructured, but these are not unsolvable; further, increasing fertility rates, commonly proposed as a solution, will only worsen the problem.

The most commonly used variant of the dependency ratio—defined as the ratio of the number of people aged ≥65 to the number people aged 15–64, is projected to increase from 16% in 2019 to 28% in 2050 (267). But this 75% increase can be misleading because it does not fully represent the number of “dependents” relative to the working population. First, workforce participation of people ≥65 years has been increasing in countries with an aging population such as USA and Japan, especially in those with the highest number of years of education (268, 269). Second, people aged ≥65 are not necessarily economically dependent. Volunteering in old age is a sizable economic sector; a study in Canada proposed that even with conservative estimates of hourly wage, the sector was worth 2 billion US dollars in 2008 (270). Third, ignoring the cost savings associated with fewer children <15 years old artificially inflates the dependency ratio (271).

Fears of an associated economic decline are unsubstantiated; investing in the health, training, and education of workers—especially older, experienced workers—in fact increases human capital, effectively making the workforce more productive (245). Concerns regarding labor shortages are also unfounded. There is no basis for an expected penury of working-age people for countries experiencing low population growth or even decline—the question reverts instead to inadequate immigration policies that limit or deny the movement of capable, working-age people from elsewhere to fill local demand. But if immigration is used to increase population growth *per se* (*cf.* fill labor vacancies), the concomitant increase in resource use and emissions resulting from immigrants increasing their *per capita* consumption rates upon successful migration to higher-income nations (271, 272) contribute to growing environmental damage.

While there will inevitably be economic and fiscal challenges accompanying aging populations (245, 246), their solutions rely more on wise policy responses, such as redesigning pension financing, investing in education to enlarge the effective workforce, and a delayed retirement age to promote higher income taxes, which subsequently improves healthcare (245). It is telling that few academic papers provide support for envisaged catastrophic consequences of population decline and aging populations (241); almost all papers in the field acknowledge existing policy implementations that successfully address these challenges. Unfortunately, misinformed and alarmist arguments against a sustainable global population remain mainstream tropes (240).

## 4 Discussion

### 4.1 Avoiding the risks associated with overpopulation

After discussing the risks associated with high population size and the reasons why the global population has already overshot the planet’s carrying capacity, we come to our central question: how do we prevent the worst-case scenario from occurring? First, we must consider our current economic model and its role in determining sustainable pathways forward.

The vicious cycle of population increase exacerbated by anthropogenic climate change, leading to even more climate change, and population growth, is a phenomenon not experienced equally globally. For example, the impact of climate change on food systems will affect everyone, but disadvantaged groups such as women, older adults, children and women in low-income households, Indigenous peoples, minorities, and smallholders, will be disproportionately burdened with malnutrition, livelihood loss, and rising costs exacerbating the cycle of existing inequalities (2). The principal drivers of anthropogenic climate change are also unequal—half of consumption-related emissions are generated by only 10% of people globally (273). Children are the most affected group, yet are not autonomous and must rely on the actions of adults, so it is incumbent on us to be their voice and protect their future.

Fulfilling a “safe and just space for all” (247) therefore requires empowering women, improving health and wellbeing for women and their children, and increasing economic prosperity (247)—actions that conveniently all lower fertility rates (274). Falling fertility is indicative of economic development, with delayed childbirth and fewer children consequences of education and career goals, and because of increased access to family planning. In many ways, lower fertility rates observed in developed countries are an indicator of female autonomy, empowerment, and equity. But women in low- and middle-income countries face multiple barriers to family-planning needs, as do an increasing number of women in high-income countries when laws and politics encroach on individual options. While the direct causality of education and fertility is unclear (275) (see section 3.3.4), there is a strong relationship between the years of maternal schooling and the probability of her children surviving (276–278). Given the most important, broad-scale determinant of reduced fertility is lower child mortality (165), the benefits are clear.

Improving women’s rights positively affects economic development (279, 280); therefore, addressing social and economic disparities are essential actions for nations to create a more just future. Indeed, the *Global Burden of Diseases Study 2019* found overwhelming evidence that social and economic development is highly correlated with positive health outcomes, and proposed prioritization to improve the status of women, expanding access to education, and stimulating economic growth through policies and strategies (281). Put simply, a healthy population is a productive population; increasing access to education accumulates human capital and improves productivity. Improving the status of women kick-starts the process of human-capital accumulation, because maternal education both directly and indirectly affects their children’s educational attainment (282, 283).

Persistently low fertility in high-income nations cannot be attributed solely to economic stressors, unemployment, lack of progressive public policies, or popular trends (e.g., postponing reproduction) (226, 284), because the phenomenon has persisted for too long and become a structural aspect of the developed world (244). For example, the demographic transition that occurred in Europe led to greater reproductive efficiency, meaning that before the transition, women spent ~70% of their adult lives bearing and rearing children (285). The corollary was that post-transition, women were massively liberated from “wasting investments” on children who ultimately died (286). Sustained high fertility and rapid population growth therefore impede sustainable development, counter to policies adopted by some countries such as Russia to prioritize population growth (233).

Policy discussions regarding overpopulation are beset by ideologies that underlie competing perspectives (287–289). Many governments attempt to boost their economic and political advantage by promoting population growth to overwhelm their less-populous neighbors (287), and there exists an unquestioned “wisdom” that has evolved over the course of human evolution that more people equates to doing better, because it meant more food, more capability, and better defenses (290). In contrast, rapid and unsustainable population growth hinders nation-level development, with China’s one-child policy an extreme example of a country limiting population growth to boost economic development (291). There has been a long-held consensus that incorporating policies and programs to reduce high fertility in developing countries is a pathway to economic development (292–294). Many have also labeled those who identify population growth as an existential challenge as “racist” and socially irresponsible for “blaming” low-income nations for overpopulation (295–297); hence, there is a reticence to engage in emotionally charged debates on the topic (287, 289). Constructive discussion on overpopulation is further inhibited because of concerns of perceived “population control” related to past abuses arising from autocratic measures to limit fertility. Providing women and men the opportunity to determine their family size free of any form of coercion cannot be deemed “population control”—rather, it is an important human right that has been neglected. In particular, empowering women—especially disadvantaged women—to make decisions about when and how many children they have, will have positive impacts on their lives and the lives of their children, and is a proven path to development.

## 5 Conclusion

The many benefits associated with lower population growth and size are unassailable, especially given the necessity of mitigating the severity of climate change over the coming centuries. But achieving “optimal” human population sizes will require major social changes that are embedded within appropriate social-cultural-ecological contexts while simultaneously respecting planetary boundaries (39, 298, 299). While we conclude that smaller human populations benefit the most people, we emphasize that we are not advocating an end to childbirth. Rather, we join the globally progressive voice of promoting the empowerment of girls and women worldwide through ethical and practical solutions to determining their own fertility. Unfortunately, neocolonial attitudes still obfuscate the links between population and environmental degradation in low- and middle-income nations, so traction for quality family planning in these fastest-growing regions has stalled. We also emphasize that determining family size should not be left to women alone; men also need to be educated adequately and provided with contraceptive options to allow them to promote prosperous and just outcomes for their family. The problems of overpopulation we outline here will not be addressed entirely through family planning and education, as beneficial as these are. Working to increase child health and implementing policies that addresses food security and climate change will also help to reduce population growth further, bringing about many corollary benefits to human societies.

## Author contributions

CS: Conceptualization, Data curation, Investigation, Methodology, Writing – original draft. MJ: Conceptualization, Data curation, Funding acquisition, Investigation, Methodology, Validation, Writing – original draft, Writing – review & editing. LW: Data curation, Investigation, Resources, Writing – review & editing. QB: Resources, Validation, Writing – review & editing. NP: Methodology, Validation, Writing – review & editing. PS: Conceptualization, Funding acquisition, Investigation, Resources, Supervision, Writing – original draft, Writing – review & editing. CB: Conceptualization, Formal analysis, Investigation, Methodology, Resources, Visualization, Writing – original draft, Writing – review & editing.
